# Aggregation-Induced Polarization (*AIP*): Optical Rotation Amplification and Adjustment of Chiral Aggregates of Folding Oligomers and Polymers

**DOI:** 10.3389/fchem.2022.962638

**Published:** 2022-08-12

**Authors:** Yao Tang, Sai Zhang, Ting Xu, Qingkai Yuan, Jia-Yin Wang, Shengzhou Jin, Yu Wang, Junyi Pan, Isaac Griffin, Daixiang Chen, Guigen Li

**Affiliations:** ^1^ Department of Chemistry and Biochemistry, Texas Tech University, Lubbock, TX, United States; ^2^ Institute of Chemistry and BioMedical Sciences, School of Chemistry and Chemical Engineering, Nanjing University, Nanjing, China; ^3^ Continuous Flow Engineering Laboratory of National Petroleum and Chemical Industry, Changzhou University, Changzhou, China

**Keywords:** chiral aggregates, multilayer folding chirality, multilayer folding oligomer, aggregation-induced polarization (AIP), multilayer folding polymer

## Abstract

The phenomenon of aggregation-induced polarization (AIP) was observed showing optical rotation amplification and adjustment. The relationship between optical rotations of chiral aggregates of multilayered chiral folding oligomers and polymers with water% in THF (*f*_w_) has been established accordingly. New multilayered chiral oligomers were synthesized under the asymmetric catalytic systems established by our laboratory recently. These products were well-characterized by UV-vis, NMR, and MALDI-TOF spectra. Absolute stereochemistry (enantio- and diastereochemistry) was assigned by comparison with similar asymmetric induction by the same catalyst in our previous reactions. The present AIP work can serve as a new tool to determine chiral aggregates, especially for those that cannot display emission. AIP would also complement AIE-based CPL since AIP serves as a new tool providing enhanced right- or left-hand polarized lights with individual wavelengths. It will find many applications in chemical and materials science in the future.

## Introduction

The study of chirality and chiral targets has become increasingly important in science and technology because of the requirements of desired chemical, biomedical, and physical properties of advanced materials (Wang et al., 1983; Taniguchi et al., 2011; Krautwald et al., 2013; Bryliakov, 2020; Zhao et al., 2020; Zhang and Kürti, 2021). In the past several decades, a significant progress has been made in controlling optical properties related to radar, lasers, optical fibers, and wireless fiber telecommunications (Huang et al., 2018; Feng et al., 2019; Xu et al., 2019; Shi et al., 2020; Liu et al., 2021; Oki et al., 2021; Zheng et al., 2022). Among these properties are circular light polarization and optical rotation for evaluating the performance of chiral targets. It is well-known that the optical fields rotate at a constant rate in a plane during circular light polarization when the waves travel (Desmarais, 1997; Singh, 2015). When waves transmit through a solution containing chiral objects, two types of rotations occur, that is, the field is rotated toward either the right-hand or left-hand direction, called right- and left-circular polarization, respectively. Left- versus right-circularly polarized lights can be generated directly by chiral molecules, nanoaggregates, and soft matters. The optical and rotational abilities of these chiral objects are anticipated to play an important role for the design and synthesis of advanced materials and their applications. In fact, the research on circularly polarized luminescence (CPL) materials has attracted much attention due to their potential to serve as displaying and optical storage devices, probes, sensors, etc. (Shi et al., 2020; Liu et al., 2021; Zheng et al., 2022).

Meanwhile, aggregation-induced emission (AIE) has grabbed widespread attention since 2001 when Tang and coworkers coined this concept based on their discovery of organic molecules displaying strong fluorescence in aggregation states (Huang et al., 2018; Feng et al., 2019). The origin of AIE was made possible by restricting intramolecular motions (rotation and vibration) which limit or avoid aggregation-caused quenching (ACQ) of conventional luminophores. During our ongoing projects on multiple-layered 3D folding chirality, we found that chiral aggregates of folding monomers, oligomers, and polymers displayed strong AIE by gradually increasing water fractions (*f*_w_) in THF-H_2_O cosolvents (Wu et al., 2019; Liu et al., 2020; Wu et al., 2020; Wu et al., 2021a; Wu et al., 2021b; Tang et al., 2022b; Jin et al., 2022; Tang et al., 2022; Wang et al., 2022). Interestingly, we also found these polymers showed AIE curves bounding when *f*_w_ was increased to 70%, which contrast with the observation of their monomer derivatives (Wu et al., 2021b; Tang et al., 2022; Wang et al., 2022). This big jump is attributed to the suppression of molecular motion in the polymer matrix, indicating that our chiral multilayer framework resulted in effective soft aggregates in THF-H_2_O co-solvents.

Since the AIE observation can conform to the formation of various chiral aggregates of multilayer chiral polymers, we envisioned that the coexistence of various chiral aggregates in THF-H_2_O solutions would lead to optical rotation enhancement or adjustment systematically. The literature search revealed nearly no documents on the X- and Y-coordinate relationship regarding aggregation-induced effects on optical rotation. In this communication, we would like to disclose our preliminary results on this study, temporally called aggregation-induced polarization (AIP).

## Result and Discussion

**FIGURE 1:**
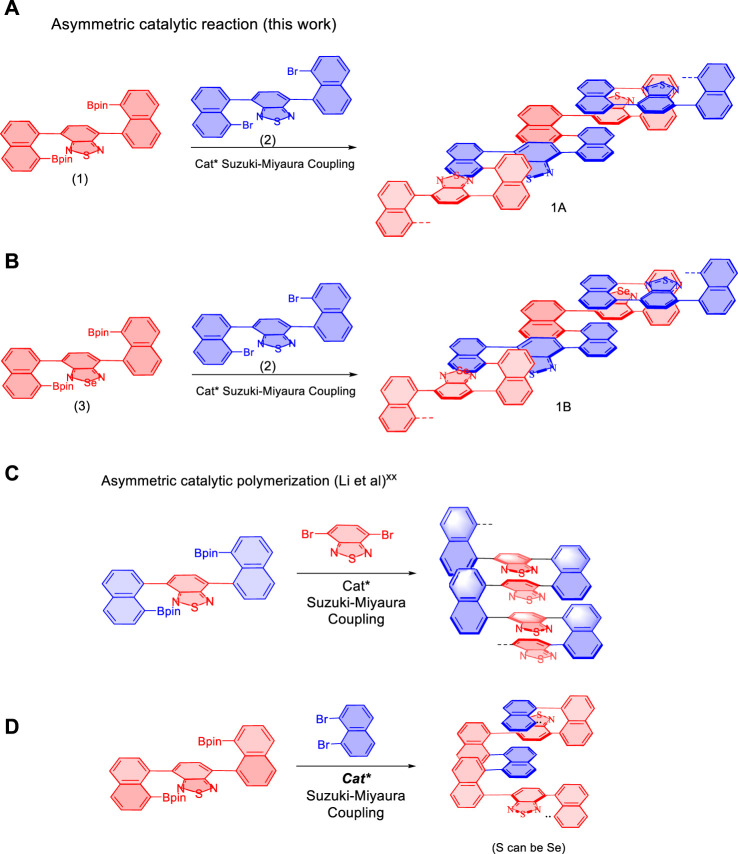
Synthesis of multilayer polymers and oligomers *via* an asymmetric approach. **(A,B)** Design and synthesis of new multilayer polymers and oligomers. **(C,D)** Reported asymmetric assembly of triple-columned and multiple-layered chiral folding polymers. Cat*: chiral catalysts.

The present AIP project was started by designing and synthesizing of new multilayer polymers and oligomers (Figures 1A,B). This work is based on the use of various new monomers which have showed success in previous asymmetric catalytic assembly of triple-columned and multiple-layered chiral folding polymers (Wu et al., 2021b; Tang et al., 2022; Wang et al., 2022) (Figures 1C,D). In the present work, new combinations of monomers were utilized by involving two pairs: 4,7-bis(8-(4,4,5,5-tetramethyl-1,3,2-dioxaborolan-2-yl)naphthalen-1-yl)benzo [c][1,2,5] thiadiazole (**1**) and 4,7-bis(8-bromonaphthalen-1-yl)benzo[c][1,2,5]thiadiazole (**2**), 4,7-bis(8-(4,4,5,5-tetramethyl-1,3,2-dioxaborolan-2-yl)naphthalen-1-yl)benzo [c][1,2,5] selenodiazole (**3**) and 4,7-bis(8-bromonaphthalen-1-yl)benzo[c][1,2,5]thiadiazole (**1**), respectively. As described in Figure 1, the resulting multilayer targets contain unique structural units of sandwich panels. The previous columns (piers) serve as decked layers holding [1,2,5]thiadiazole or [1,2,5]selenodiazole as unit cores. For potential applications of the new framework, electron transfer would occur concurrently through-space among three layers of sandwich panels and conjugation between sandwich units.

In our previous work, both C and D series exhibited intense fluorescence activity and remarkable optical properties in aggregated states, more specifically, photoluminescence in solids and aggregation-induced emission (AIE) in solutions under specific wavelength irradiation (Wu et al., 2021b; Tang et al., 2022; Wang et al., 2022). In the meanwhile, C series presents were found to display not only AIE characteristics but also reversible redox properties in electrochemical performance.

**SCHEME 1:**
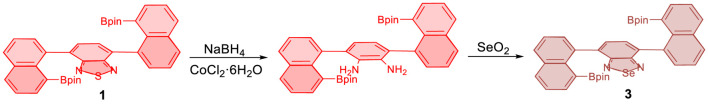
Synthesis of selenodiazole monomers.

Monomer 4,7-bis(4,4,5,5-tetramethyl-1,3,2-dioxaborolan-2-yl)benzo[c][1,2,5]thiadiazole (**1**) was synthesized by reacting bis(pinacolato)diboron with 4,7-bis(8-bromonaphthalen-1-yl)benzo[c][1,2,5]thiadiazole, which was preformed from the treatment of 4,7-bis(4,4,5,5-tetramethyl-1,3,2-dioxaborolan-2-yl)benzo[c][1,2,5]thiadiazole with 1,8-dibromonaphthalene under traditional Suzuki-Miyaura cross-coupling conditions followed by treating with bis(pinacolato)diboron (Miyaura and Suzuki, 1995; Kumar et al., 2020). Selenodiazoles (**3**) was derived from its thiadiazole counterpart through the reductive opening of thiadiazole ring by using an excess amount of sodium borohydride (4.0 equiv) in the presence of CoCl_2_·6H_2_O (10 mol%) (Scheme 1) (Neto et al., 2005; Idris et al., 2018). The resulting crude diamine product was directly subjected to the next step for the ring-closure reaction with selenium dioxide (2.0 equiv) in ethanol at 80°C (Scheme 1). The reasons for selecting thiadiazole and selenodiazole infrastructures for the present project are based on the fact that they are among the most frequently used scaffolds in polymers and materials science (Vyskocil et al., 2002; Jurok et al., 2010; Echeverri et al., 2020), and have been proven to be successful in asymmetric catalytic polymerization.

**FIGURE 2:**
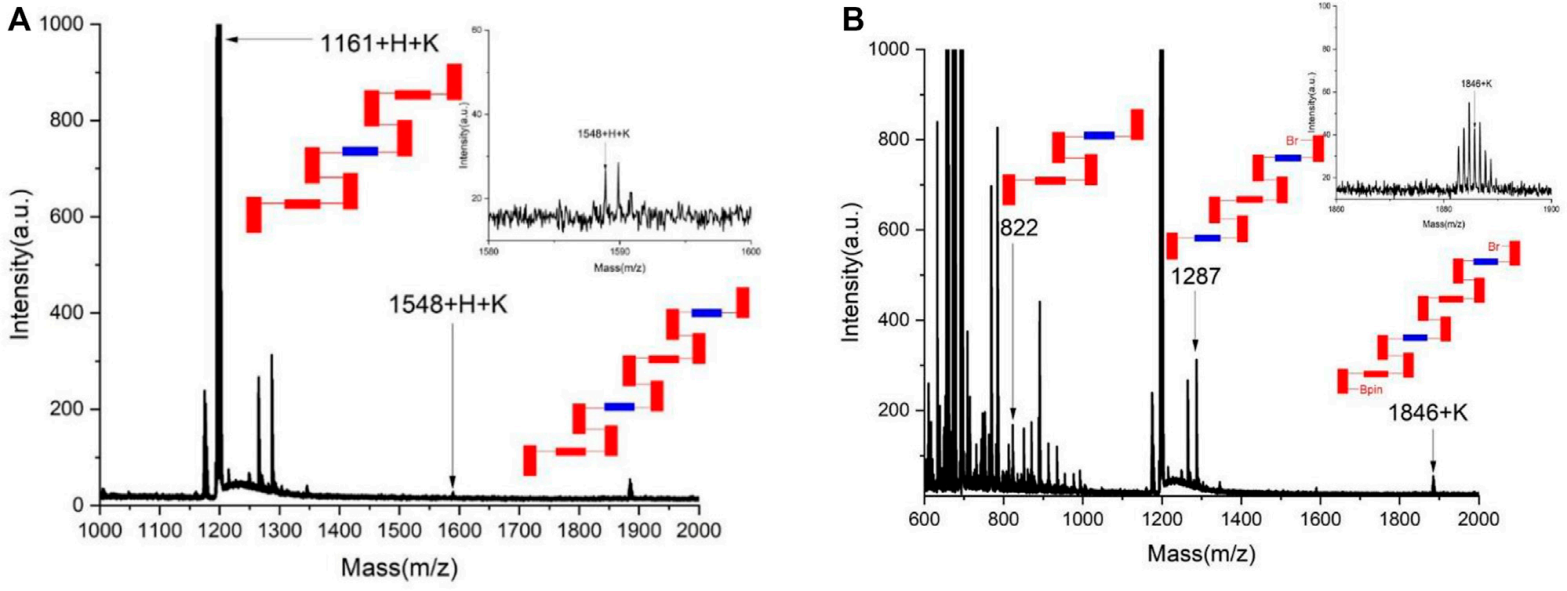
MALDI-TOF analysis of chiral oligomers 1A and 1B. **(A)** MALDI-TOF of the chiral oligomer 1A. **(B)** MALDI-TOF of the chiral oligomer 1B.

As reported previously, asymmetric polymerization was attempted under the standard catalytic system. [20-21] Pd(*S*-BINAP) Cl_2_ was utilized as the chiral catalyst in THF/H_2_O co-solvents in the presence of K_2_CO_3_ at 85°C for 6 days. Surprisingly, chiral polymers could not be well-formed under the standard condition. Instead, two sets of corresponding chiral oligomers were afforded in chemical yields of 57.7% for **1A** and 47.9% for 1B, respectively. As shown in Figure 2, MALDI-TOF indicated that both **1A** and **1B** contain up to four layers in their layered frameworks. While **1B** is attached with Br and 4,4,5,5-tetramethyl-1,3,2-dioxaborolan-2-yl moieties at each terminal of this oligomer, 1A does not contain these two moieties as shown by MALDI-TOF analysis (Figure 2).

It is challenging to purify individual oligomers for X-ray structural analysis because various species coexist in the mixture products including relevant stereoisomers. The absolute configuration of major enantiomers was assigned by comparison with the previous stereoselective control by the same catalyst of Pd(*S*-BINAP)Cl_2_. This catalyst was anticipated to give the same asymmetric induction in the present catalytic processes (Wu et al., 2021a; Wu et al., 2021b; Tang et al., 2022; Wang et al., 2022). It should be noted that the asymmetric catalytic formation of **1A** and **1B** would provide a new atropisomeric framework for controlling orientational/rotational chirality if a chiral tetrahedron center replaces one of two Bpin or Br moieties in oligomer **1B**.

**FIGURE 3:**
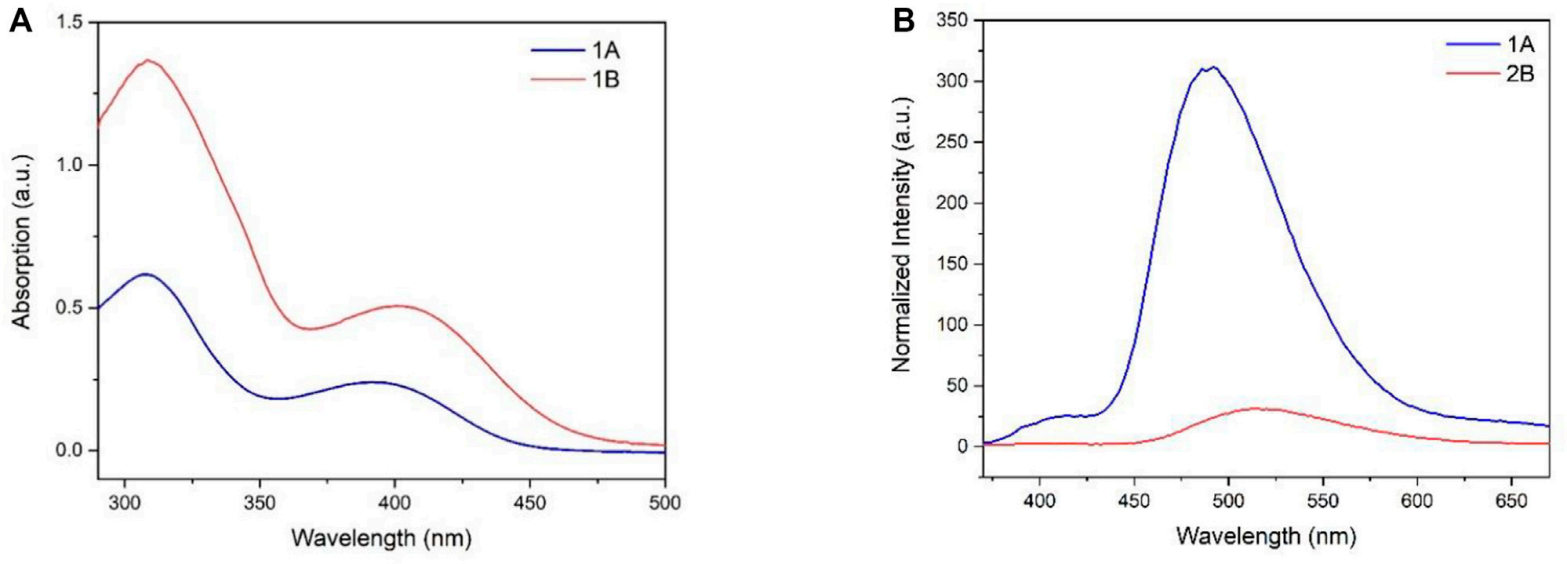
**(A)** UV/Vis absorption spectra of **1A** and **1B** in THF; c = 50 μg/ml; **(B)** Normalized fluorescent spectra of **1A** and **1B** (50 μg/ml) in THF, excitation wavelength: 350 nm.

The UV-Vis absorption of compounds **1A** and **1B** in THF was recorded (Figure 3). **1A** and **2B** exhibited similar maximum absorptions at 308 nm, and **1A** showed a board absorption band between 355 and 450 nm. However, replacing sulfur from the heterocycle on the one side of the backbone with a heavier chalcogen atom Se led to a redshift on the broadband due to the lowered energy band (10 nm for **1B** vs. **1A**). Similar behavior can also be found in the emission spectrum, where **2B** with the selenium atom shifted to the longer wavelength (520 nm, compared to **1A**). Surprisingly, **1B** with sulfur atoms on both sides of backbones exhibited six-fold stronger fluorescence intensity.

**FIGURE 4:**
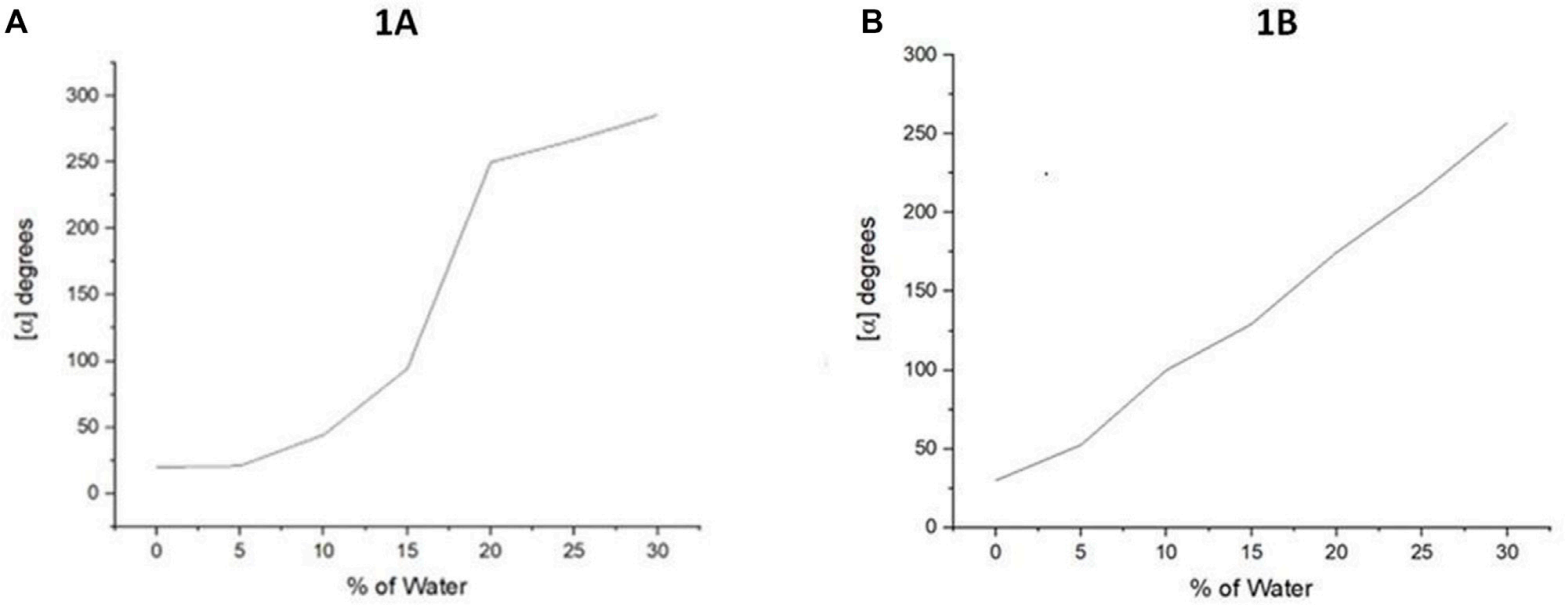
Aggregation-induced polarization (AIP) of new multilayer 3D chiral folding oligomers. **(A)** AIP of chiral oligomer **1A** in THF; *c* = 1 mg/ml. **(B)** AIP of the chiral oligomer **1B** in THF; *c* = 1 mg/ml.

After we determined their UV-vis, we attempted to determine aggregation-induced emission (AIE) for **1A** and **1B**, but we found none of them exhibited AIE. This result made it difficult to observe their aggregation in solution *via* emission spectroscopy directly. As shown in Figure 4 both **1A** and **1B** displayed positive optical rotation in THF, chloroform, and methanol, respectively. We, thus, decided to systematically investigate the relationship between their aggregation and optical rotation. This work would result in systematic polarization enhancement and adjustment simply by changing cosolvents and provide a new tool to study the chiral aggregation of chiral molecules that do not exhibit AIE.

The popularity of inexpensive polarimeters in most asymmetric synthesis labs makes conducting such an AIP study very convenient. We utilized a Rudolph polarimeter (Rudolph Research Analytical APIV/2W) to acquire optical rotation data of chiral polymers and oligomers at around room temperature with a sodium lamp as the light source. Measurements were performed in a vessel of 2 ml with concentrations (c = 1 mg/ml or 0.5 mg/ml) in THF and its cosolvents. When the fraction of water (*f*_w_) reached over 30% or 35%, the optical rotation data often showed a certain degree of instability for most solutions in this study, which would be attributed to the fact that the glass surface tension at the two ends of a vessel is enlarged.

In this measurement, the water fraction (*f*_w_) was set at the component of 5% (v/v) on the X-horizontal coordinate corresponding to specific rotation on the Y-vertical ordinate. As revealed in Figure 4 that under the standard aggregation cosolvents of THF and water, we observed a consistent relationship between the optical rotation of **1A** and **1B** with *f*_w_ by gradually increasing water fractions in the cosolvents (Figure 4). Oligomer **1A** showed a big jump when the water fraction was increased from 15 to 20%. However, its counterpart **1B** showed a nearly straight line when *f*_w_ was increased from 0 to 30%. This trend was not continued beyond 30% *f*_w_ because of their solubility and optical rotation stability displayed in the instrument. The average data on three measurements for each sample were adopted in plotting the relationship curves in AIP Figures.

**FIGURE 5:**
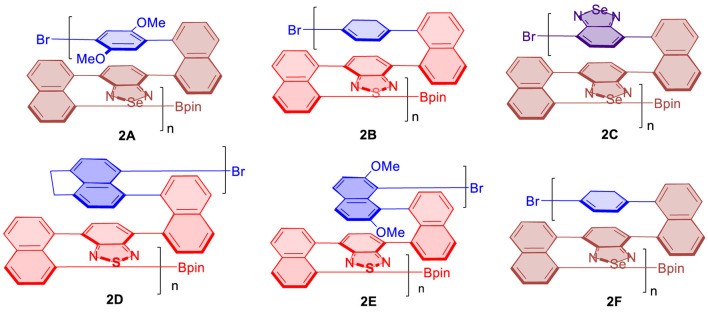
Multilayer 3D chiral polymers for the AIP study.

**FIGURE 6:**
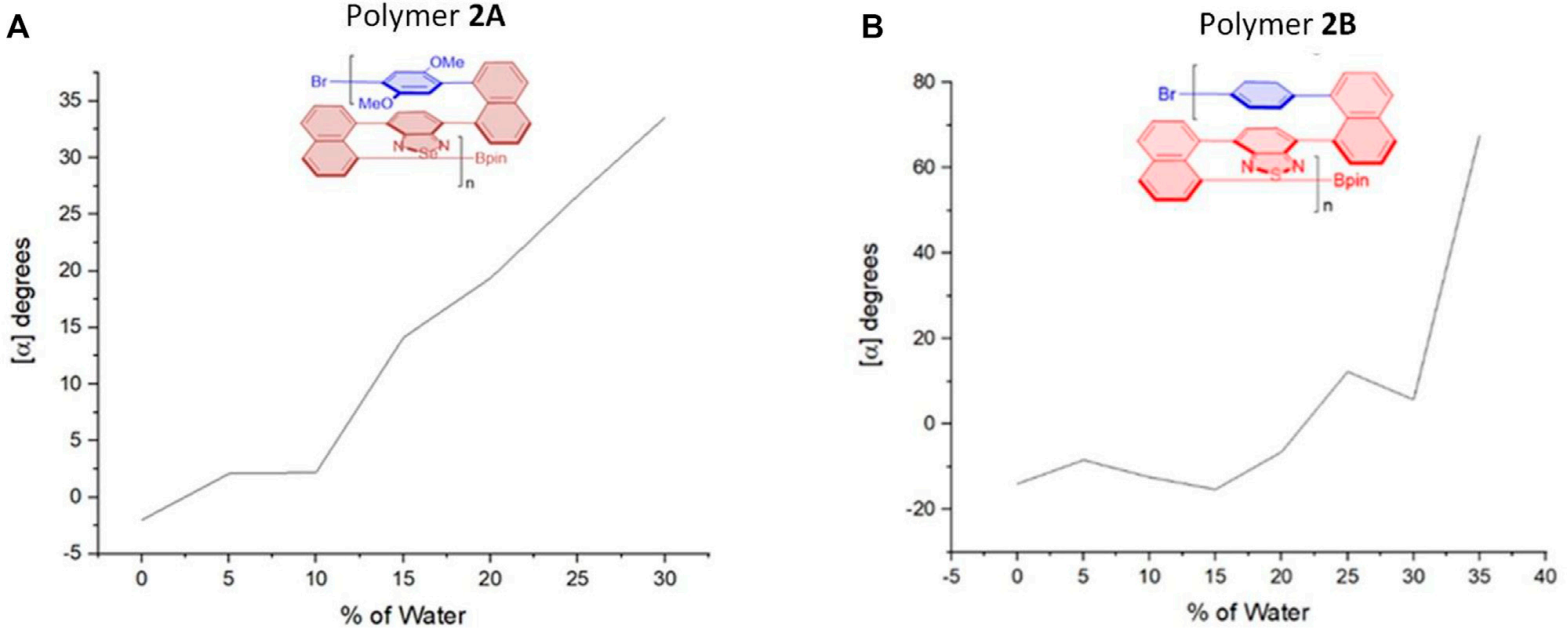
Aggregation-induced polarization (AIP) of reported multilayer 3D chiral folding polymers. **(A)** AIP of chiral folding polymer **2A** in THF; *c* = 1 mg/ml. **(B)** AIP of chiral folding polymer **2B** in THF; *c* = 0.5 mg/ml.

**FIGURE 7:**
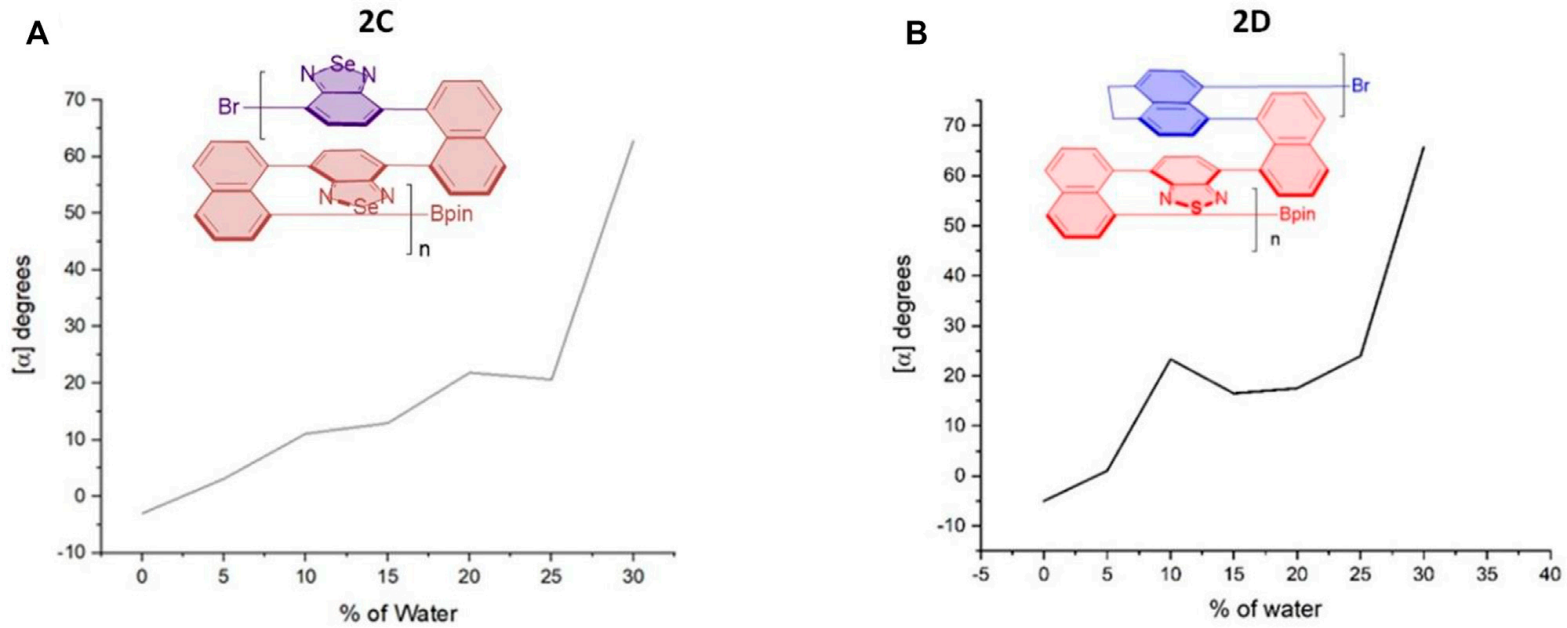
Aggregation-induced polarization (AIP) of reported multilayer 3D chiral folding polymers. **(A)** AIP of chiral folding polymer **2C** in THF; *c* = 1 mg/ml. **(B)** AIP of chiral folding polymer **2D** in THF; *c* = 1 mg/ml.

**FIGURE 8:**
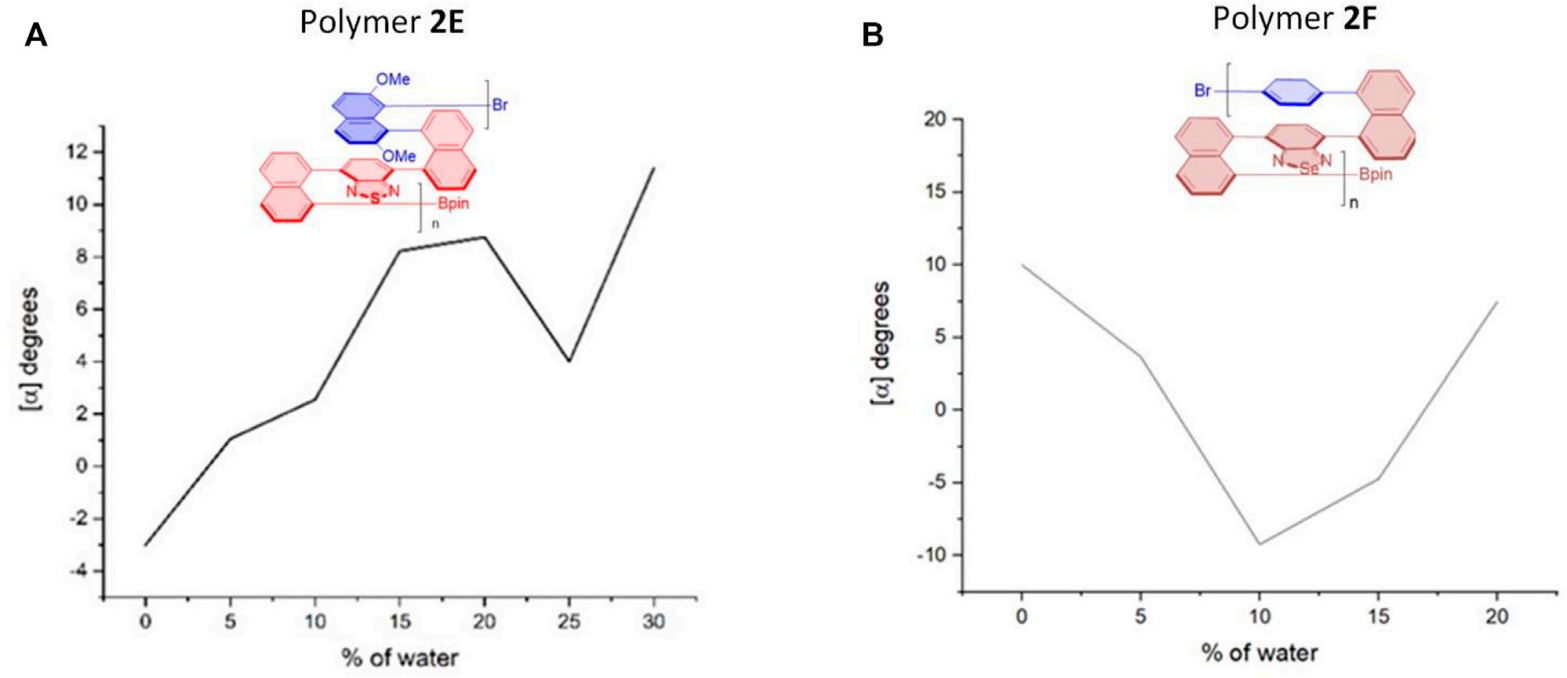
Aggregation-induced polarization (AIP) of reported multilayer 3D chiral folding polymers. **(A)** AIP of chiral folding polymer **2E** in THF; *c* = 1 mg/ml. **(B)** AIP of chiral folding polymer **2F** in THF; *c* = 1 mg/ml.

After we observed the aforementioned relationship, we checked previous multilayer chiral folding polymers (Figure 5) for the possible aggregation-induced polarization (AIP) (Wu et al., 2021b; Tang et al., 2022; Wang et al., 2022). Convincingly, we found these polymers also displayed noticeable AIP effects, albeit the resulting relationship curves appeared in different shapes (Figures 6–8). Polymer **1A** exhibited a nearly straight line similar to oligomer **1B** staying flat between 5 and 10%, but a big jump appeared when %water was increased from 10 to 15%. The optical rotation of polymer **2B** stayed negative at −6.5° until the water fraction (*f*_w_) reached 25% showing positive optical rotation data as 12.2°. There was a big jump between 30 and 35% *f*_w_ when optical rotation was enhanced from 5.7° to 67.7°, although optical rotation was slightly decreased in plot periods of 5–15%, and 25–30%, respectively.

For the case of polymer **2C**, optical rotation was started with negative data of --3.0^o^ in THF in the absence of water (*f*_w_ = 0%), but these data turned out to be positive when *f*_w_ was increased to 5%. The positive data remained afterward until the water fraction reached 30%. Similar to the case of **2B**, a big jump came out when *f*_w_ was increased from 25 to 30%. Polymer **2D** also showed an overall enhancement of optical rotation. Two big jumps of its optical rotation were observed when *f*_w_ was increased from 5 to 20% and then from 25 to 30%, respectively, although the second jump is relatively higher than the first one, which is similar to **2B** and **2C** cases. Polymer **2D** also displayed negative data in THF in the absence of water and showed positive when 5% water was added (Figure 7).

Interestingly, in the cases of polymers **2E** and **2F** (Figure 8), there are two “V” patterns in their AIP relationship curves. In the former, a smaller “V” appeared between water fractions of 20 and 30%, and a bigger “V” was observed during the whole *f*_w_ range of 0–20%. While the optical rotation appeared from a negative number at the beginning (*f*_w_ = 0%) for five cases (**2A**–**2E**), the opposite phenomenon was encountered for the case of **2F**. Polymer **2F** showed a positive optical rotation of 10.0^o^ at the beginning, but quickly dropped to –9.2° when the water fraction was increased to 10%. The optical rotation was then turned in the opposite direction back to 7.5°.

For all of the cases that were examined in this AIP work, it is very difficult to provide a mechanistic explanation at this early stage. The complicated situation was caused by the fact that the multilayer chiral oligomers and polymers exist as complexes of different aggregation sizes. Thus far, there has been no direct correlation between optical rotation and structures of complex mixtures reported in the literature to the best of our knowledge lacking empirical data to study the AIP mechanism in detail. More research turned out to be necessary regarding molecular design and computational and physical organic chemistry in the future.

When the products of oligomers (**1A** & **1B**) and polymers (**2D** & **2E**) did not display aggregation-induced emission (AIE) (Wu et al., 2021b), four other polymers, **2A**–**2C** and **2F**, exhibited remarkable AIE in their solutions, as reported in this study. All of these polymers (**2A**–**2F**) displayed the optical property of photoluminescence in solids (Wu et al., 2021b; Tang et al., 2022; Wang et al., 2022). In addition, polymers **2A**, **2B**, **2C**, and **2F** were also found to exhibit reversible redox properties in electrochemical performance (Tang et al., 2022; Wang et al., 2022).

**FIGURE 9:**
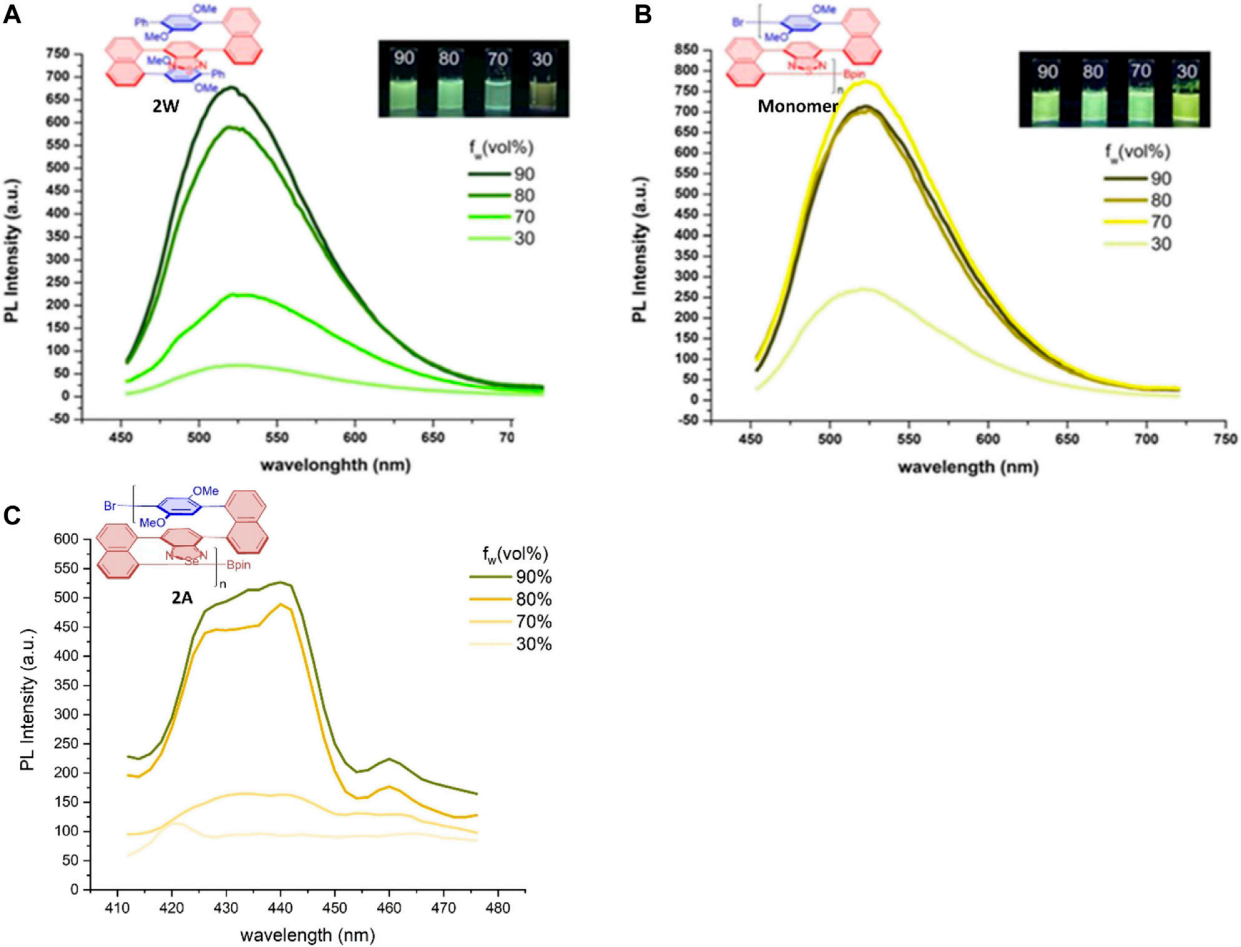
Aggregation-induced emission (AIE) of polymers and monomer. **(A,B)** PL spectra of **2W** and its monomer in THF/water mixtures with different water fractions (f_w_); c_monomer_ = 0.1 mM, c_2w_ = 50 ug/mL; λ_ex_(momomer) = 380 nm, λ_ex_ (**2W**) = 385 nm; inset: fluorescence. ^*^ **(C)** PL spectra of 2A in THF/water mixtures with different water fractions (f_w_); c2A = 50 ug/mL; λ_ex_ (2A) = 374 nm. ^*^ PL spectra of 2W and its monomer is reprinted from ref. 21. (Copyright 2021, Wiley-VCH Verlag GmbH & Co. KGaA).

It should be pointed out that the right- or left-polarized lights generated via AIP is fundamentally different from that through CPL or AIE-based CPL regarding the light sources or polarization mechanisms. In addition, the AIP spectrum shows the relationship between water fraction (*f*_w_) and optical rotation ([α]). In contrast, the AIE spectrum represents the relationship between water fraction (*f*_w_) and PL intensity. We re-plotted the relationship of AIE polymer **2W** and the PL intensity of its monomer with water fractions (*f*_w_) based on the maximum PL intensity of each curve (Figure 9), and a similar AIE effect can also be found in polymer **2A**. As described in Figure 9, although that the relationships of these properties of **2E** with water fraction (*f*_w_) are generally along the same direction, its AIP adopted water fractions (*f*_w_) until 35% since the glass surface tension at the two ends of the vessel is enlarged during its AIP measurements. Photographs of monomer & 2WE in THF/water system. Reprinted with permission from ref. 21. (Copyright 2021, Wiley-VCH Verlag GmbH & Co. KGaA).

It is noteworthy that AIE and CPL spectra belong to the molecular spectrum since their polarized left- or right-hand light beams are directly emitted from chiral molecules or chiral aggregates, which belong to emission spectroscopy analysis, and usually give a broad range of wavelengths. In contrast, AIP belongs to transmission spectroscopy analysis since its polarized left- or right-hand light beams are generated by transmitting through the solutions containing chiral compounds (about interaction with externally polarized lights). Its light beams are originally generated from metal or metal filament cycle lamps (external light sources, e.g., Na lamps) with individual wavelengths. A single wavelength of light beams (weak laser) is transmitted inside polarimeter equipped with a filter and detector, which would be treated as indirect light sources. Therefore, AIP would provide an alternative tool for generating left- or right-hand light beams in which the strength of the laser is controlled by atomic spectroscopy sources such as Na lump. We believe that the present AIP work is essential not only for material science in seeking challenging physical properties, but also for chemical reactions and syntheses by taking advantage of aggregate soft matters including chiral ones. Therefore, they would complement each other in research and applications in aggregation science.

## Conclusion

In summary, we have established the relationship between the optical rotation of chiral aggregates of multilayered chiral folding oligomers and polymers with water% in THF. The typical aggregation cosolvent systems resulted in optical rotation amplification and adjustment, defined as aggregation-induced polarization (AIP). This phenomenon was discovered during the design and synthesis of novel multilayered chiral oligomers and then found to exist in THF-H_2_O systems of triple-column/multiple-layer chiral folding polymers. The multilayered chiral oligomers were efficiently synthesized under the asymmetric catalytic systems established by our laboratory recently. These products were well characterized by UV-vis, NMR, and MALDI-TOF spectra. Absolute stereochemistry (enantio- and diastereochemistry) was assigned by comparison with similar asymmetric induction by the same catalyst in our previous reactions. The AIP work can serve as a new tool to determine chiral aggregates, especially those that cannot display emissions. Therefore, AIP and AIE are anticipated to complement each other. In addition, AIP can also complement AIE-based CPL since AIP serves as a new tool providing enhanced right- or left-hand polarized lights with individual wavelengths. It is anticipated to find many applications in chemical and materials sciences in future.

## Data Availability

The original contributions presented in the study are included in the article/Supplementary Material, and further inquiries can be directed to the corresponding author.
