# Supplementary material for: Aggregation-Induced Polarization (AIP): Optical Rotation Amplification and Adjustment of Chiral Aggregates of Folding Oligomers and Polymers
Source: Front Chem. 2022 Aug 12;10:962638. doi: 10.3389/fchem.2022.962638 (PMC9413080; doi:10.3389/fchem.2022.962638)
Supplement: Supplementary file 1 [file DataSheet1.pdf]

# Electronic Supporting Information for

# Aggregation-Induced Polarization (*AIP*): Optical Rotation Amplification/Adjustment of Chiral Folding Oligomers and Polymers

Yao Tang,[1,†] Sai Zhang,[1,†] Ting Xu,[2,†] Qingkai Yuan,[1] Jia-Yin Wang,[2] Shengzhou Jin,[2] Yu Wang,[2] Junyi Pan,[2] Isaac Griffin[1#] Daixiang Chen,[3] and Guigen Li[1,2,]*

[1]*Department of ~~Chemistry~~ and Biochemistry, Texas Tech University, Lubbock, Texas 79409-1061, USA.*
[2]*Institute of Chemistry and BioMedical Sciences, School of Chemistry and Chemical Engineering, Nanjing University, Nanjing, 210093, China.*
[3]*Continuous Flow Engineering Laboratory of National Petroleum and Chemical Industry, Changzhou University, Changzhou, Jiangsu 213164, China.*

*Correspondence should be addressed to Guigen Li; guigen.li@ttu.edu

†These authors contributed equally to this work.

# Undergraduate participant.

## Table of Content

1. General Information ........ S2
2. Synthetic Procedures ........ S3
3. MALDI-TOF Analysis ........ S6
4. NMR Spectra ........ S8
5. References ........ S14

## 1. General Information

Unless otherwise stated, all reactions were magnetically stirred and conducted in oven-dried glassware in anhydrous solvents under Ar. Solvents and liquid reagents, as well as solutions of solid or liquid reagents were added directly or via syringes, or micropipette. Cooling baths were prepared in Dewar vessels filled with ice/water (0 °C). Heated oil baths were used for reactions requiring elevated temperatures. Solvents were removed under reduced pressure at 40-65 °C using a rotavapor. All given yields for small molecules are isolated yields of chromatographically and NMR spectroscopically materials.

All commercially available chemicals were used as received without further purification. Solvents as follows: MeOH, EtOH, toluene, EtOAc, DCM, dioxane, hexane, acetone and THF were used without further purification.

The $^{1}H$ and $^{13}C$ NMR spectra were recorded in $CDCl_3$ on 400 MHz and 100MHz instruments with TMS as internal standard. For referencing of the $^{1}H$ NMR spectra, the residual solvent signal ($\delta$ = 7.26 for $CDCl_3$) were used. In the case of the $^{13}C$ NMR spectra, the signal of solvents ($\delta$ = 77.06 ± 0.03 for $CDCl_3$) were used. Chemical shifts($\delta$) were reported in ppm with respect to TMS. Data are represented as follows: chemical shift, multiplicity (s = singlet, d = doublet, t = triplet, m = multiplet), coupling constant (*J*, Hz), and integration. MALDI-TOF analyses were carried out using an ABI/MDS SCIEX 4800 Mass Spectrometer with HABA matrix. UV-Vis spectra were collected on an Agilent 8453 UV-Visible Spectroscopy system. Fluorescence spectra were collected by Agilent Technologies Cary Eclipse Fluorescence Spectrophotometer G9800A and Eclipse ADL program.

## 2. Synthetic Procedures

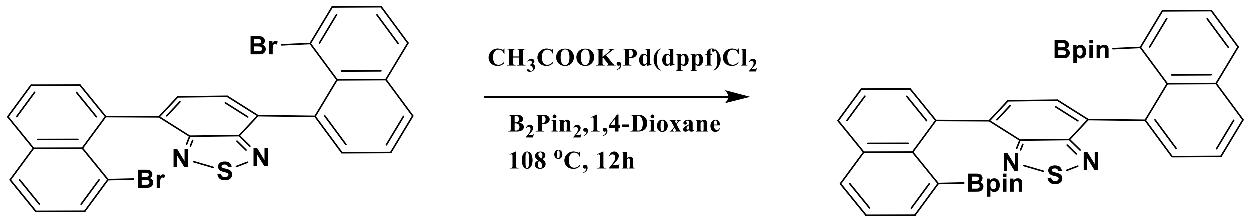


1

**4,7-bis(8-(4,4,5,5-tetramethyl-1,3,2-dioxaborolan-2-yl)naphthalen-1-yl)benzo [c][1,2,5] thia-diazole (1)**

To an oven-dried 50 mL round flask equipped with a condenser, 4,7-bis(8-bromonaphthalen-1-yl)benzo[c][1,2,5]thiadiazole **1-1** (294 mg 1.0 mmol), bis(pinacolato)- diboron (559 mg, 2.2 mmol), Pd(dppf)Cl2 (73 mg, 0.10 mmol), and KOAc (588 mg, 6.0 mmol) were dissolved in 20 mL 1,4 dioxane. The whole system was degassed under vacuum and backfilled four times untiled filled with argon. The resulting reaction mixture was stirred in the inner environment at 108 °C for 12 hours. After the reaction was completed, dioxane was removed by rotavapor. The residue was dissolved with DCM, washed with water and brine, and dried with $MgSO_4$. The organic solvent was evaporated by rotavapor and purified by column chromatography (Hexane/EtOAc = 10/1) to obtain pure product as yellow solid **1** (0.413 g, 65%).

$^1$H NMR (400 MHz, $CDCl_3$) δ 7.96 (dd, *J* = 8.2, 1.3 Hz, 2H), 7.91 (dd, *J* = 8.1, 1.1 Hz, 2H), 7.80 (dd, *J* = 6.8, 1.3 Hz, 2H), 7.76 (dd, *J* = 7.2, 1.2 Hz, 2H), 7.59 – 7.54 (m, 4H), 7.48 (dd, *J* = 8.2, 6.8 Hz, 2H), 0.79 (d, *J* = 9.2 Hz, 12H), 0.62 (s, 12H).

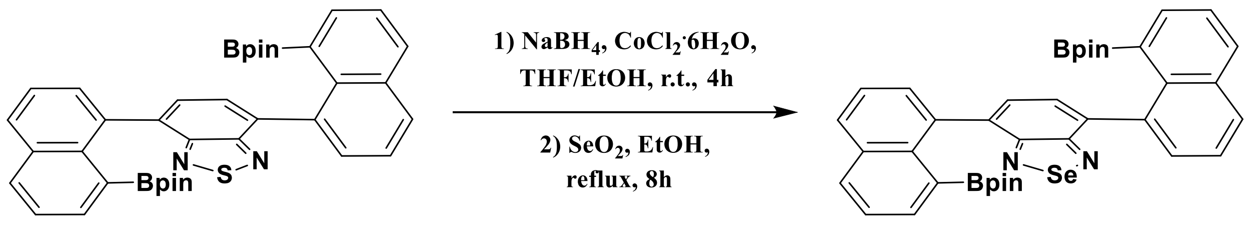


1

3

**4,7-bis(8-(4,4,5,5-tetramethyl-1,3,2-dioxaborolan-2-yl) naphthalen-1-yl) benzo [c][1,2,5] selenadiazole (2)**

In an oven-dried 50 mL round flask, Diboron 1 (640 mg, 1.0 mmol) was dissolved in a 20 mL THF/EtOH mixture (1:1). Followed up by adding $NaBH_4$ (152 mg, 4.0 mmol) and $CoCl_2{\cdot}6H_2O$ (38 mg, 0.1 mmol), and the resulting reaction was stirred vigorously at room temperature for 4 hours. TLC monitored the reaction until all starting material was consumed completely. The excessed $NaBH_4$ was quenched by $NH_4Cl$, and the organic solvent was removed by rotavapor. The residue was dissolved with 20 mL ethyl acetate, and the aqueous phase was washed with 20 ml ethyl acetate two more times. The organic phase was washed with brine and dried over $MgSO_4$. The crude product was used for the next step without further purification.

To an oven-dried 50 mL round flask, $SeO_2$ (222 mg, 2.0 mmol) was added to the ethanol solution of the former product in an argon environment. The reaction mixture was stirred at 80 $^oC$ for 8 hours. After the reaction was completed, the reaction mixture was cooled to room temperature and extracted with DCM. The organic solvent was removed by rotavapor, and the residue was purified by column chromatography using Hexane/EtOAc (8/1) as an eluent to get the pure product as yellow solid **2** (0.32 g, 47%).[1]

$^1$H NMR (400 MHz, $CDCl_3$) δ 7.93 (ddd, *J* = 20.8, 8.2, 1.1 Hz, 4H), 7.83–7.74 (m, 4H), 7.58–7.53 (m, 2H), 7.50–7.45 (m, 2H), 7.42 (s, 2H), 0.84 (s, 12H), 0.73–0.60 (m, 12H).

Typical polymerization procedure for Oligomers **1A** and **1B**

Take Oligomer **1A** as an example for synthesis.

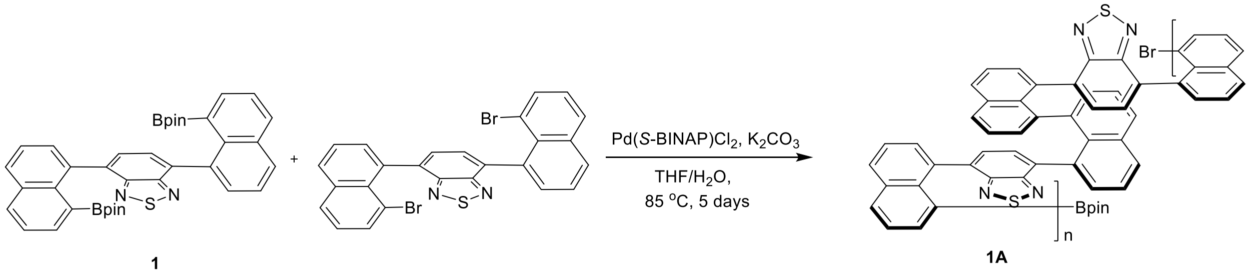


**1** **1A**

To an oven dried 50 mL round bottle flask under argon, **1** (128.0 mg, 0.2 mmol, 1 equiv), **3** (58.0 mg, 0.2 mmol, 1 equiv), Pd(*S*-BINAP)$Cl_2$ (8.0 mg, 0.01mmol, 0.05 eq) and $K_2CO_3$ (110.0 mg, 0.8 mmol, 4 equiv) were dissolved into 9.5 mL THF and 1.5 mL $H_2O$, equipped with an air condenser. The bottle flask was degassed under vacuum and backfilled with argon 6 times. It

was then heated at 85 °C for 6 days. After reaction completed, the mixture was cooled to room temperature and slowly poured reaction solution into MeOH/6N HCl (25 mL/5 mL). The precipitated solution was stirred for 0.5 h at room temperature, then recovered by filtration through a Buchner funnel. The solid was separately washed with 2N HCl (10 mL) and $H_2O$ (10 mL) for one more time, and dried at oven at 56 °C for overnight. Yellow solid (130 mg, 84.7% yield, $[\alpha]_D^{RT}$= +20 (*c* =0.1, THF), MALDI-TOF$_{max}$ (m/z = 1548). $^1H$ NMR (400 MHz, $CDCl_3$) δ 8.10-6.50 (m, Ar-H). $^{13}C$ NMR (101 MHz, $CDCl_3$) δ 134-122 (m, Ar-C).

Synthesis of Oligomer **1B**

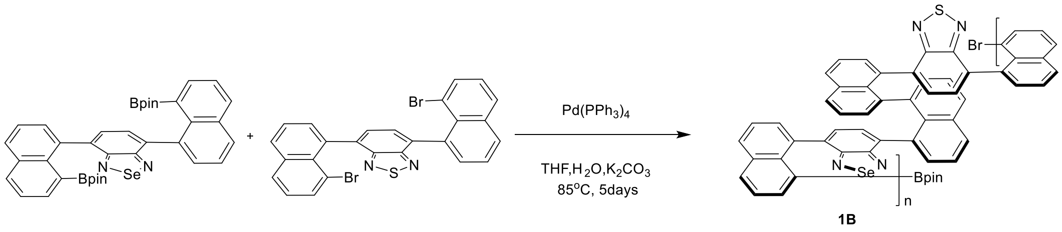


Oligomer **1B** was obtained as a yellow solid (78 mg, 47.9% yield, $[\alpha]_D^{RT}$= +30 (*c* = 0.1, THF, MALDI-TOF$_{max}$ (m/z =1846). $^1H$ NMR (400 MHz, $CDCl_3$) δ 8.00-5.30 (m, Ar-H). $^{13}C$ NMR (101 MHz, $CDCl_3$) δ 131-121 (m, Ar-C).

## 3. MALDI-TOF Analysis

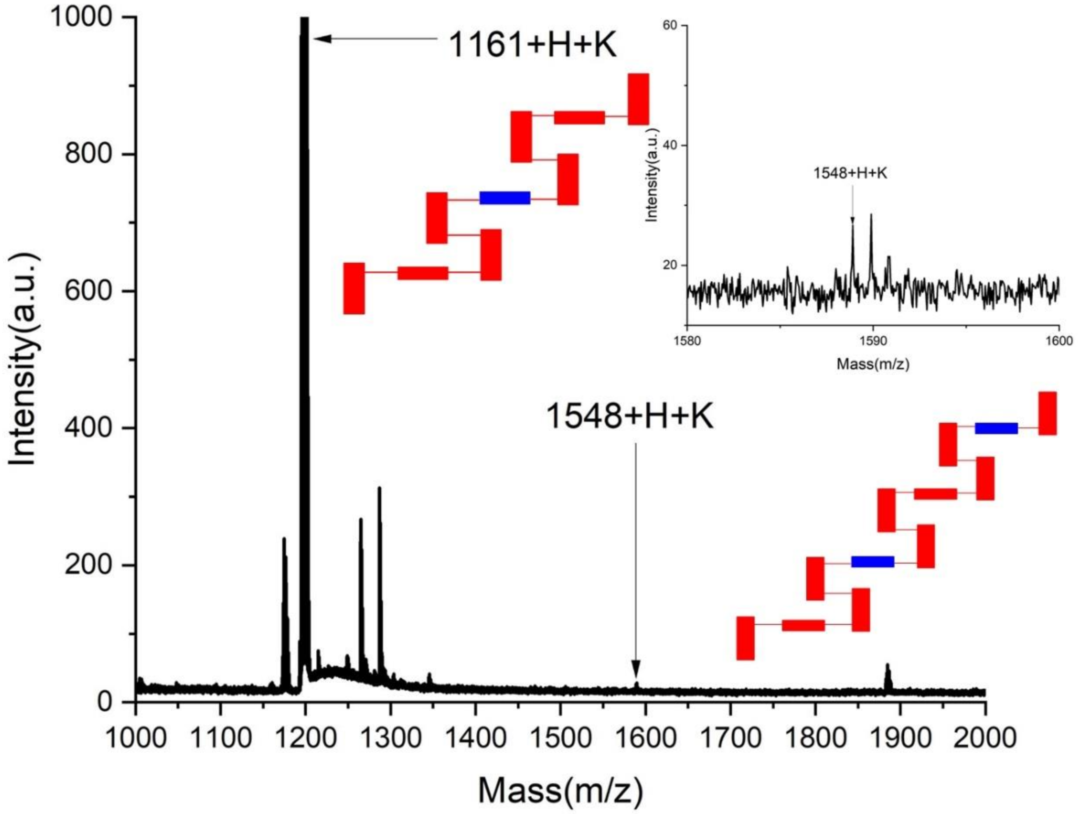


**Fig. S1** MALDI-TOF of chiral oligomer **1A**.

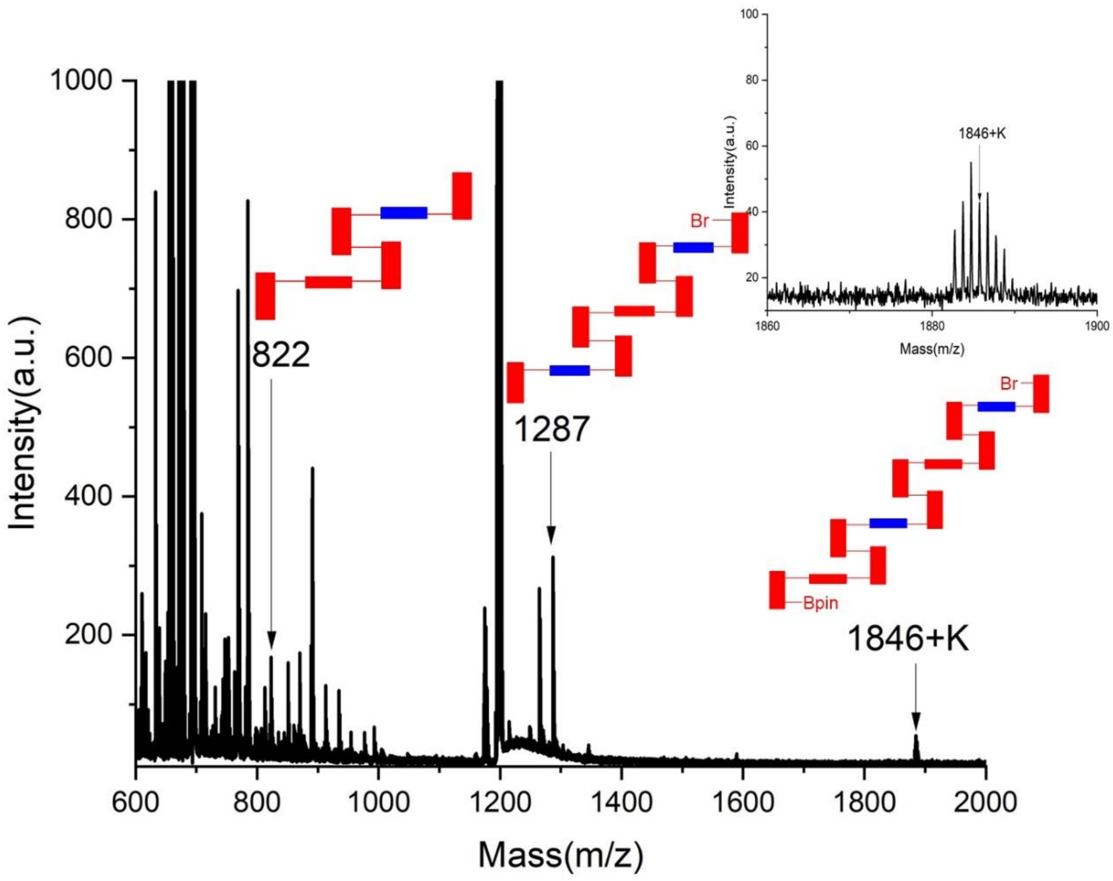


**Fig. S2** MALDI-TOF of chiral oligomer **1B**.

## 4. NMR spectrum

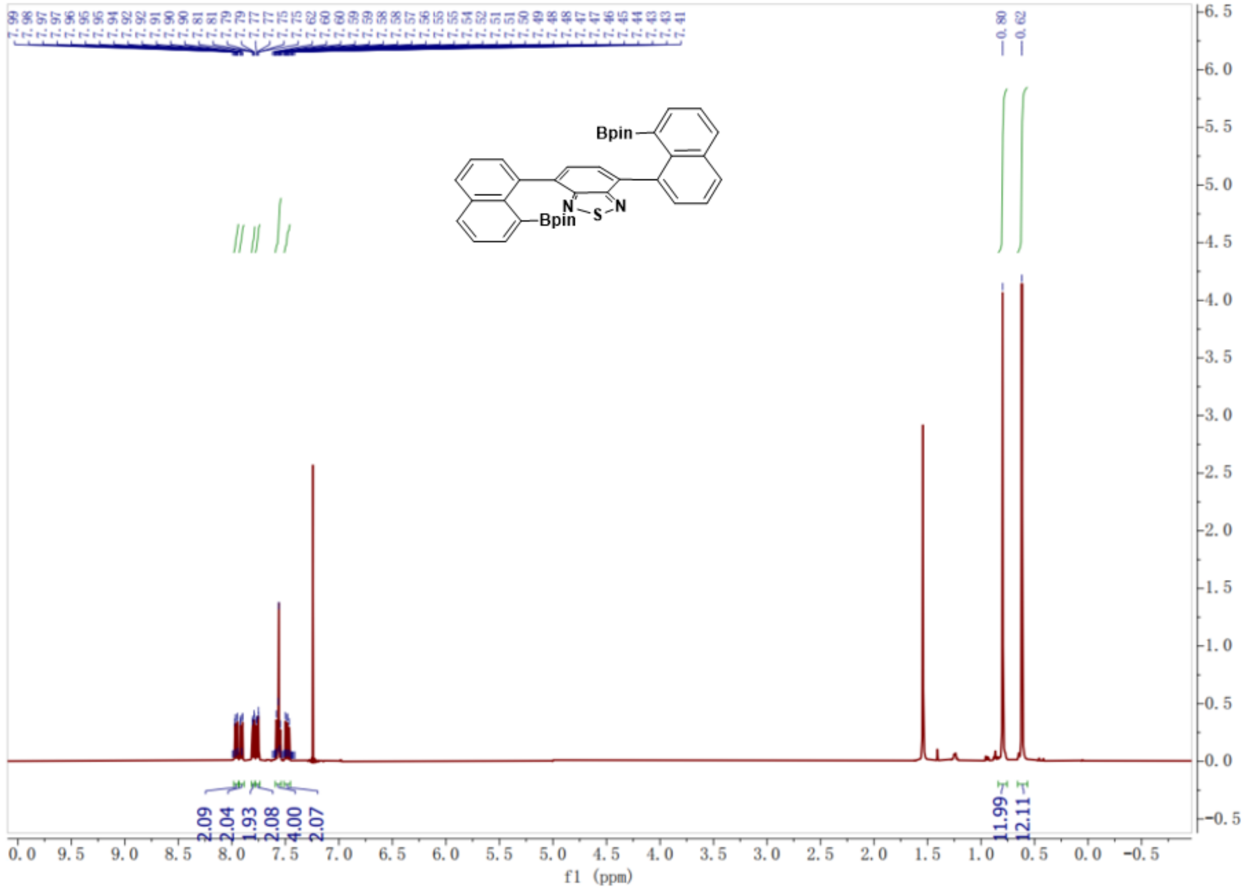

**Fig. S3** $^{1}$H NMR spectrum of monomer **1**.

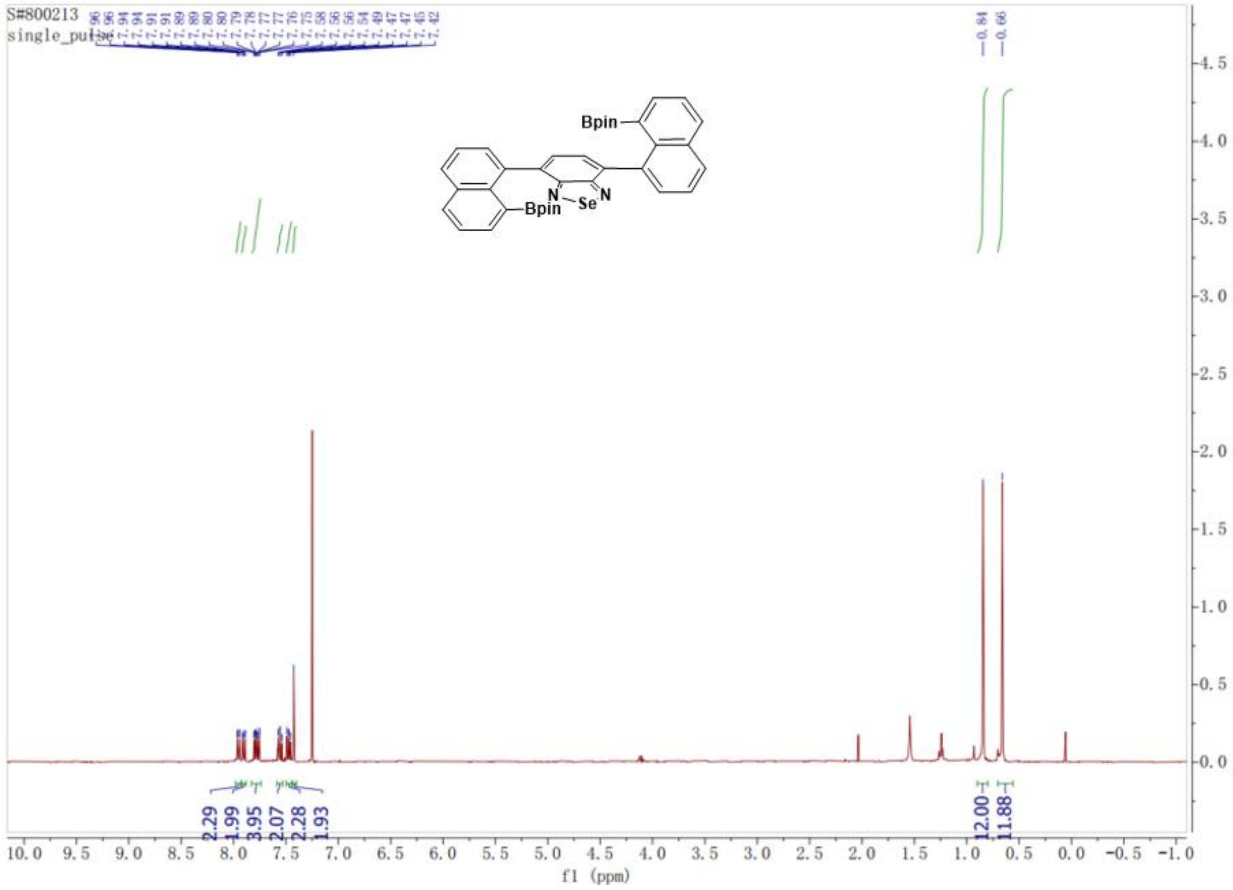

**Fig. S4** $^{1}$H NMR spectrum of monomer **3**.

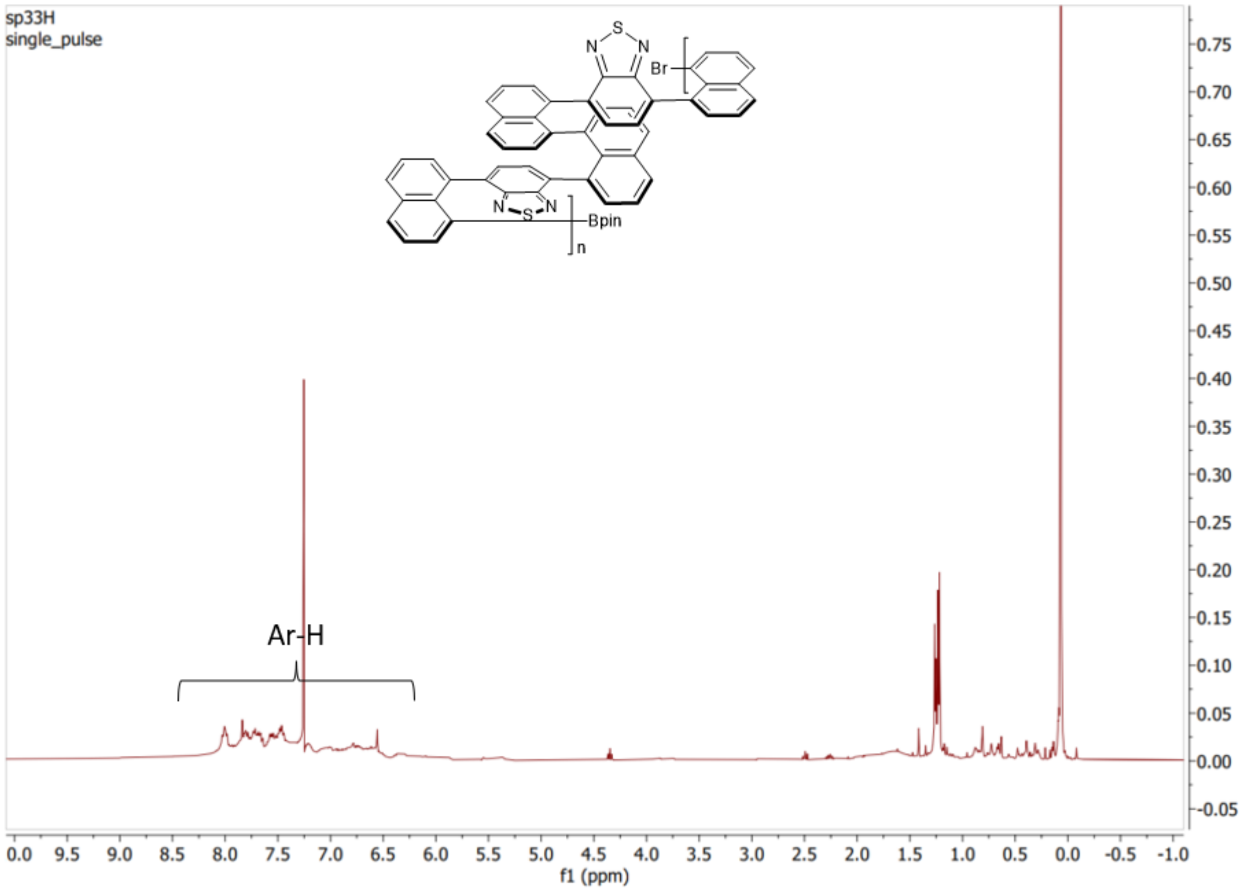


**Fig. S5** $^1$H NMR spectrum of oligomer **1A**.

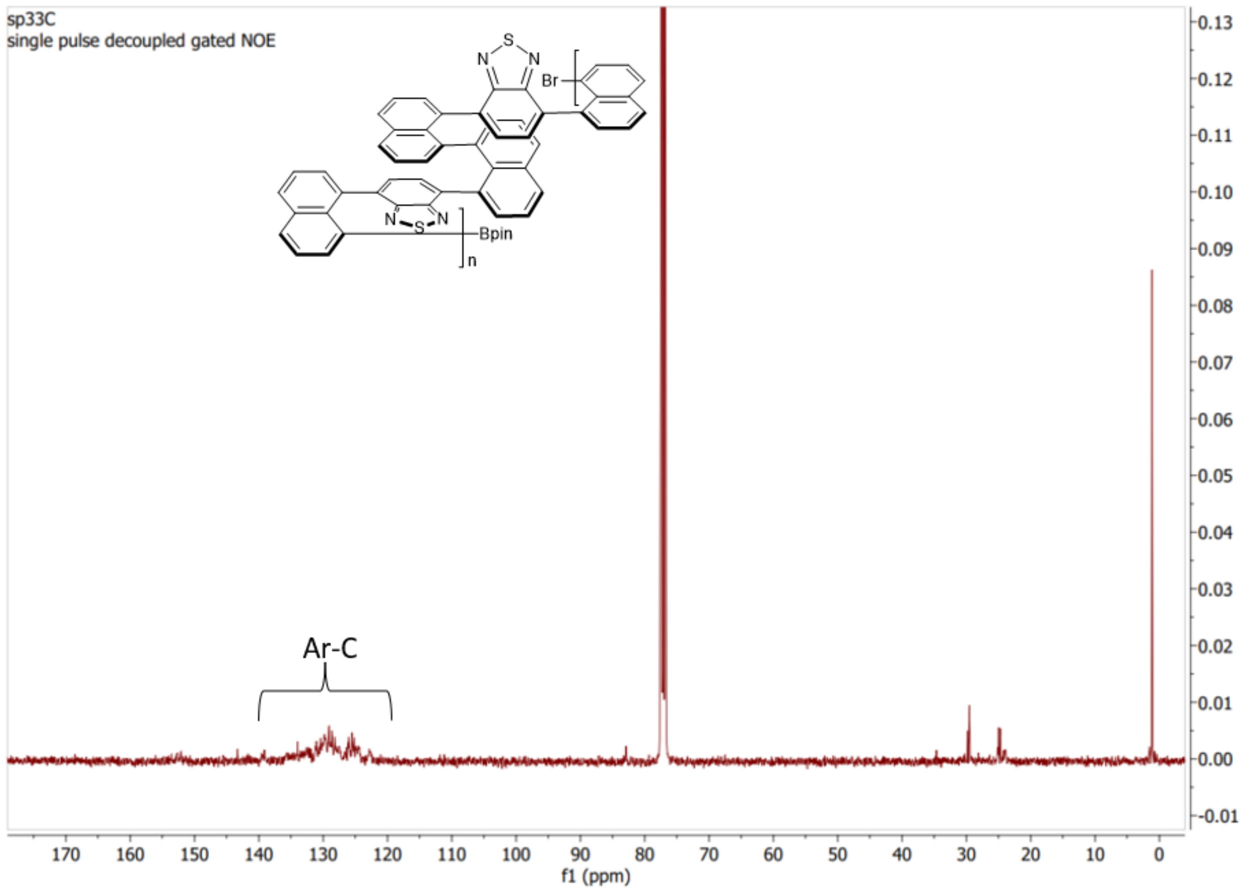


**Fig. S6** $^{13}$C NMR spectrum of oligomer **1A**.

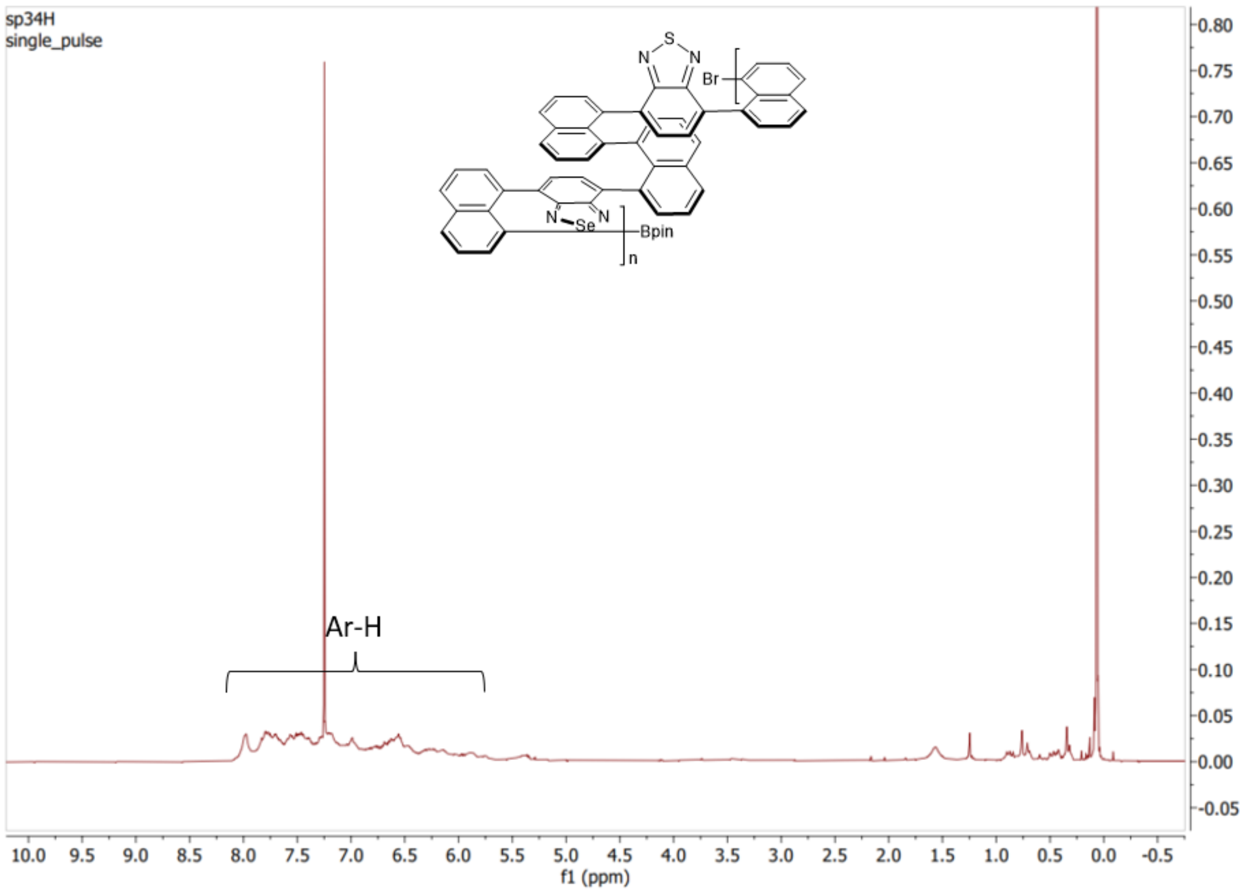


**Fig. S7** $^{1}$H NMR spectrum of oligomer **1B**.

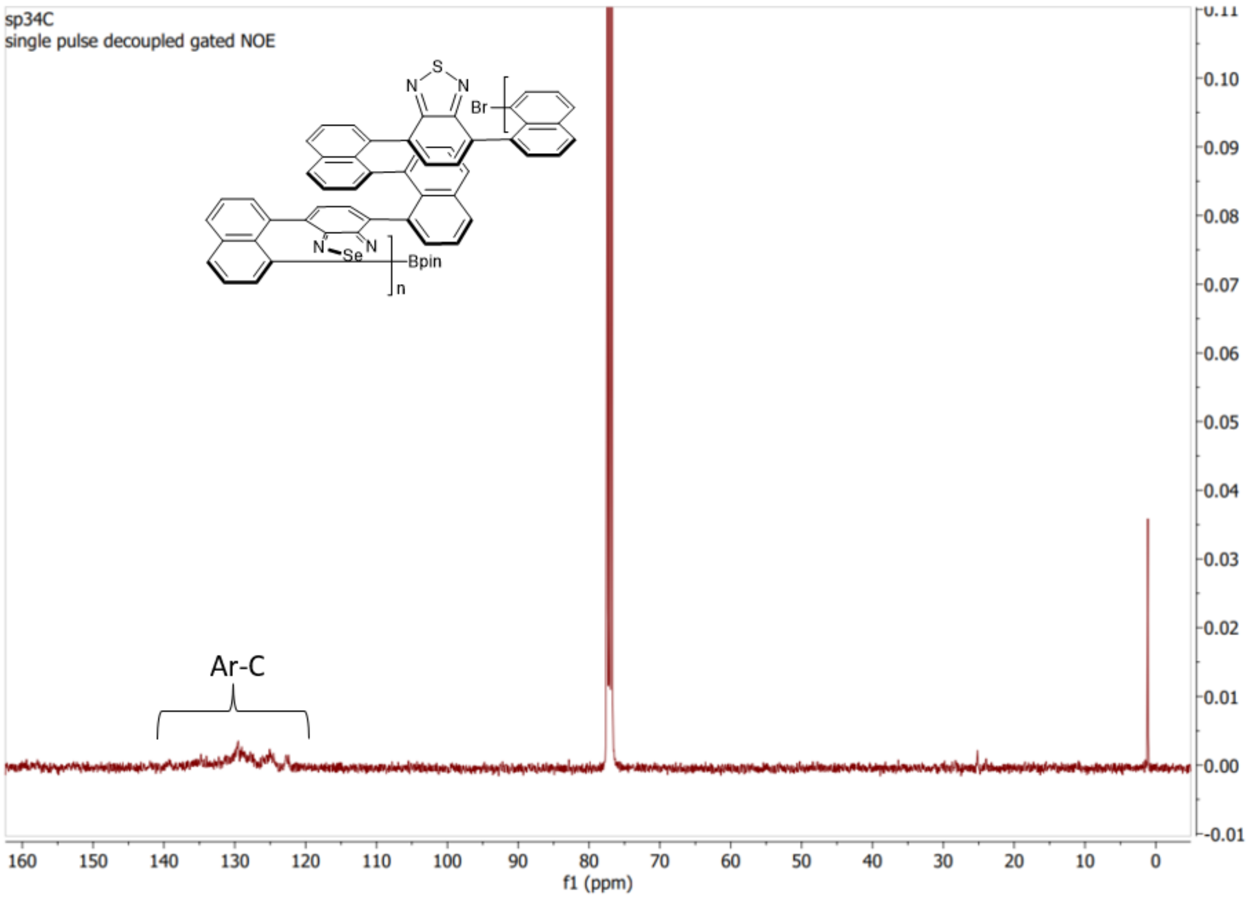


**Fig. S8** $^{13}$C NMR spectrum of oligomer **1B**.

## 5. Reference

(1) J.-Y. Wang, Y. Tang, G.-Z. Wu, S. Zhang, H. Rouh, S. Jin, T. Xu, Y. Wang, D. Unruh, K. Surowiec, Y. Ma, Y. Li, C. Katz, H. Liang, W. Cong and G. Li, *Chemistry Eur. J.*, 2022, **28**, e202104102.
